# Effects of dietary selenium deficiency and supplementation on liver in grazing sheep: insights from transcriptomic and metabolomic analysis

**DOI:** 10.3389/fvets.2024.1358975

**Published:** 2024-06-19

**Authors:** Xiwei Jin, Lingbo Meng, Zhi Qi, Lan Mi

**Affiliations:** School of Life Sciences, Inner Mongolia University, Hohhot, China

**Keywords:** deficiency, metabolomic, selenium, sheep, supplementation, transcriptomics

## Abstract

**Background:**

Mineral elements play a crucial role in supporting the life activities and physiological functions of animals. However, numerous studies have revealed that in some geographical areas and certain grazing situations, grazing livestock frequently suffers from mineral element deficiencies due to the loss of mineral elements from grassland forages, such as selenium (Se). To shed fresh light on this issue, this study aims to investigate the impact of dietary Se deficiency and supplementation on the liver of grazing sheep in these challenging conditions.

**Method:**

This study involved 28 grazing Mongolian Wu Ranke sheep with an average body weight of about 32.20 ± 0.37 kg, which were divided into the Se treatment group and the control group. The Se treatment group was fed with the low-Se diet for 60 days and then continued to be fed with the high-Se diet for 41 days. The liver concentration of minerals, transcriptomic analysis, and untargeted metabolomic analysis were conducted to assess the impact of Se deficiency and supplementation on the liver of grazing sheep.

**Results:**

Dietary Se deficiency and supplementation significantly reduced and elevated liver concentration of Se, respectively (*p* < 0.05). Gene functional enrichment analysis suggested that dietary Se deficiency might impair protein synthesis efficiency, while Se supplementation was found to enhance liver protein synthesis in grazing sheep. *AGAP1*, *ERN1*, *MAL2*, *NFIC*, and *RERG* were identified as critical genes through the weighted gene correlation network analysis, the quantitative real-time polymerase chain reaction, and the receiver operating characteristic curve validation that could potentially serve as biomarkers. Metabolomics analysis revealed that dietary Se deficiency significantly reduced the abundance of metabolites such as 5-hydroxytryptamine, while dietary Se supplementation significantly elevated the abundance of metabolites such as 5-hydroxytryptophan (*p* < 0.05).

**Conclusion:**

Integrative analysis of the transcriptome and metabolome revealed that dietary Se deficiency led to reduced hepatic antioxidant and anti-inflammatory capacity, whereas Se supplementation increased the hepatic antioxidant and anti-inflammatory capacity in grazing Wu Ranke sheep. These findings provide new insights into the effects of dietary Se deficiency and supplementation on the liver of grazing sheep, potentially leading to improved overall health and well-being of grazing livestock.

## 1 Introduction

Sheep are regarded as a highly significant livestock species in terms of global food production, as they offer valuable resources such as fur, meat, and goat’s milk for humans (1, 2). Trace essential minerals, serving as essential cofactors and enzyme components in diverse biological processes, play a pivotal role in the immune, oxidative, and energy metabolism of sheep. It mainly includes zinc (Zn), copper (Cu), cobalt (Co), magnesium (Mn), selenium (Se), etc. These micronutrients are crucial for the growth and development of sheep (3, 4). Nevertheless, numerous studies have revealed that grazing livestock in predominantly grazing areas suffer from mineral intake deficiencies. This is mainly caused by the loss of mineral elements from the soil, which limits forage from providing grazing livestock with enough mineral nutrients (5). Livestock husbandry is a predominant sector in the Inner Mongolia Autonomous Region of China, serving as the primary industry and source of revenue for local herders (6). Our previous studies have also identified mineral deficiencies in Inner Mongolia grazing sheep, particularly Se (7).

Se is an essential trace mineral element for sheep, with antioxidant, anti-inflammatory, anti-carcinogenic, and anti-parasitic properties (8). It is one of the most important essential trace elements in livestock production. As a constituent of 25 selenoproteins, Se plays a significant role in reproduction, thyroid hormone metabolism, and antioxidant defense (9). Ruminants exhibit lower Se absorption efficiency compared to monogastric animals and poultry, resulting in frequent occurrences of Se deficiency without additional supplementation in the diet (10). The initial report of Se deficiency in ruminants dates back to 1957, with the main symptoms being muscle tremors, difficulty swallowing, and increased heart rate, known as “white muscle disease” (11). Moreover, dietary Se deficiency negatively impacts reproductive efficiency and mammary health (11). Transcriptomic analyses reveal that dietary Se deficiency resulted in excessive mitochondrial fission and decreased expression of the *Mfn2* and *Opa1* in calf liver, as well as increased apoptosis and necroptosis in human uterine smooth muscle cells, and down-regulation of 19 Se proteins, including GPX1, GPX2, and GPX3 (12, 13). Our previous findings also demonstrated that dietary Se deficiency impaired liver, myocardium, and pancreas function in grazing sheep, which could be appropriately alleviated by supplementing with Se (7). Much research has illustrated that the liver serves as a primary organ for Se storage (10). However, there are fewer studies on the effects of Se on the liver of grazing sheep. It is still unclear how selenium deficiency and supplementation impact gene expression and metabolite fluctuations in the liver.

Transcriptomics and metabolomics are valuable tools for investigating changes in gene expression and metabolite profiles in animals. Therefore, the experiment divided 28 grazing Mongolian Wu Ranke sheep into control group and Se treatment group. The Se treatment group was fed with deficient multi-nutrient salts diet for 60 days and then fed with supplement multi-nutrient salts diet for 41 days. The liver weight, concentration of essential mineral elements, transcriptomics, and metabolomics were analyzed after deficient and supplement treatments to investigate the effects of Se deficiency and supplementation on the liver of grazing sheep. The aim of this study is to provide fresh light on the hepatic impact of dietary Se deficiency and supplementation, while also providing data guidance for the sensible mineral supplementation of grazing sheep.

## 2 Materials and methods

### 2.1 Animal ethics

All animal procedures were approved and conducted in strict accordance with the requirements set out by the Inner Mongolia University Animal Care and Use Committee (IMU-2020-sheep-040).

### 2.2 Experimental design

A total of 28 4-month-old female grazing Wu Ranke sheep, with an average body weight of about 32.20 ± 0.37 kg, were purchased from Abaga Banner, Xilin Gol League, Inner Mongolia Autonomous Region, China. All sheep were housed individually and fed with crushed oats, natural grass, and multi-nutrient salts according to the National Research Council’s (NRC, 2007) suggestion (7).

**Figure 1:**
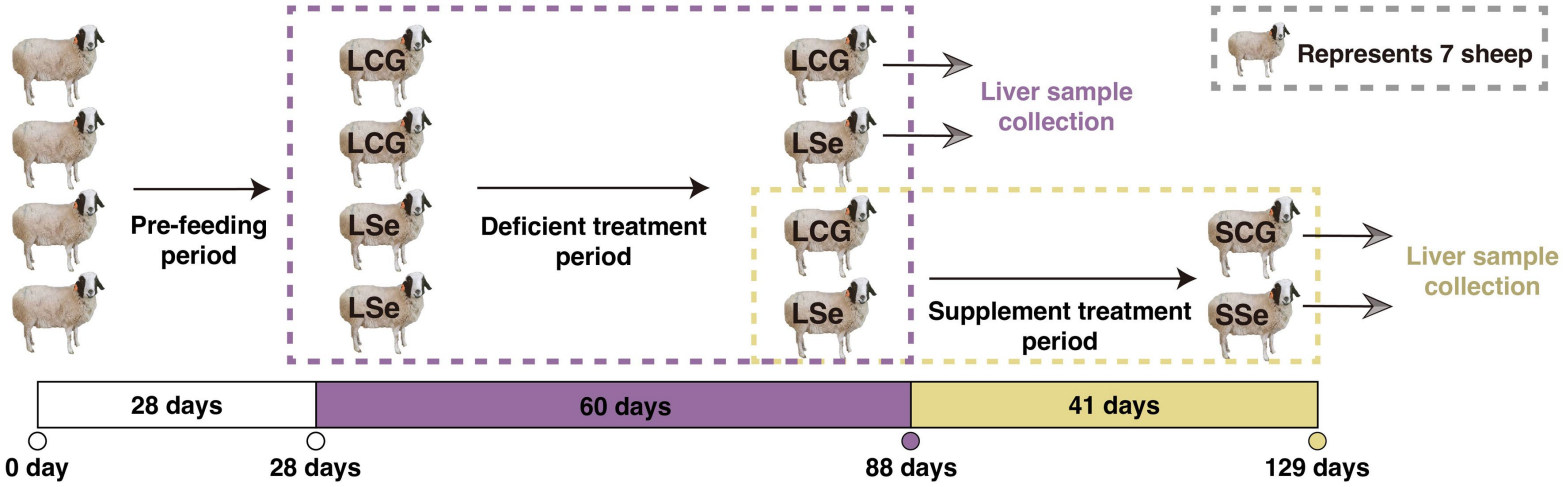
Feeding experimental design of the selenium treatment group and control group in 28 grazing Wu Ranke sheep. LSe, the selenium deficient group; LCG, the control group of the selenium deficient treatment period; SSe, the selenium supplement group; SCG, the control group of the selenium supplement treatment period.

The feeding experiment design is shown in Figure 1. Only crushed oats and natural grass were fed to grazing Wu Ranke sheep during the 28 days pre-feeding period. The 28 Wu Ranke sheep were randomly separated into the Se deficient group (LSe) and the control group of the Se deficient treatment period (LCG) at the end of the pre-feeding period. LSe and LCG were fed with Se deficient multi-nutrient salt and standard multi-nutrient salt for 60 days, respectively. Subsequently, 7 sheep were chosen at random from each group to be slaughtered. The remaining 7 sheep in the LSe and LCG groups were provided with Se supplement multi-nutrient salt and standard multi-nutrient salt, respectively. They were then slaughtered after 41 days, forming the Se supplement group (SSe) and the control group of the Se supplement treatment period (SCG). The formulation of multi-nutrient salts for each group is shown in Supplementary Table S1 and nutrient content in oats and natural grass is shown in Supplementary Table S2.

### 2.3 Sample collection

The Wu Ranke sheep were fasted for 12 h before slaughter. Liver samples were collected at the end of the Se deficient treatment period (day 88) and the Se supplement treatment period (day 129) during the feeding period. The liver samples were weighed for fresh weight, followed by snap-freezing in liquid nitrogen and subsequent storage at −80°C for future laboratory analysis.

### 2.4 Detection of essential mineral elements in liver

The liver samples were digested using a microwave digester (REVO, Labtech, Beijing, China), and the digestion procedure can be found in Supplementary Table S3. Analysis of the concentrations of 10 essential mineral elements in the liver, including phosphorus (P), sulfur (S), potassium (K), calcium (Ca), manganese (Mn), iron (Fe), cobalt (Co), copper (Cu), zinc (Zn), and selenium (Se), was performed using a TXRF spectrometer equipped with a molybdenum (Mo) X-ray tube (S4 T-STAR, Bruker Nano GmbH, Berlin, Germany). Each sample was measured for 300 s, and the results for all liver minerals were calibrated against a standard curve to ensure accuracy (7).

### 2.5 Transcriptomic analysis

#### 2.5.1 RNA extraction and Illumina sequencing

Total RNA was extracted and purified from each liver sample using the TRIzol reagent (Ambion, TX, United States) as per the manufacturer’s protocol (14). The RNA Nano 6,000 Assay Kit was applied to the Bioanalyzer 2,100 system (Agilent Technologies, CA, United States) to assess the RNA quality. Five sheep livers were randomly selected from each group to construct cDNA libraries using the NEBNext^®^ Ultra™ RNA Library Prep Kit for Illumina^®^. The cDNA libraries from 20 Wu Ranke sheep livers (5 sheep per group) for sequencing using the Illumina Novaseq platform and 150 bp paired-end reads were generated (15).

#### 2.5.2 Quality control, reads mapping to the reference genome, and quantification of gene expression level

The raw data was first assessed for quality using FastQC software.^1^ The result provided information about the base composition of the sequence and the quality of the corresponding sequence (16).

The reference genome and gene annotation files were downloaded for sheep (Oar_rambouillet_v1.0) from the Ensembl website.^2^ Salmon software was used to construct the reference genome index, and separately align the reads to the reference genome (17). Gene expression of each transcript was calculated and expressed as transcripts per kilobase million reads (TPM) (18).

#### 2.5.3 Identification of differentially expressed genes

Differentially expressed genes (DEGs) were identified between LSe and LCG, as well as SSe and SCG, using the DESeq2 R package. The significance of the DEGs was determined based on criteria of |log2foldchange| ≥ 1 and a false discovery rate (FDR) < 0.05 (the *p*-value adjusted by the Benjamini-Hochberg method) (19).

#### 2.5.4 Weighted gene co-expression network analysis

The Weighted Gene Co-expression Network Analysis (WGCNA) was a bioinformatic analysis technique that efficiently examines the relationship between genes and phenotypic data. The co-expression networks for the LSe, SSe, LCG, and SCG were constructed using DESeq2-normalized gene expression data by the WGCNA R package. The highly co-expressed gene modules were inferred using WGCNA for 9,253 genes in the Wu Ranke sheep of these groups. The PickSoftThreshold was utilized to determine the optimal soft threshold for selecting and validating gene co-expression modules. To identify modules based on topological overlap, the matrix data was converted into an adjacency matrix and subsequently clustered. Clustering dendrograms were generated by computation of module eigengene (ME) and merging related modules in the tree based on ME. In this experiment, the hepatic concentration of Se served as the phenotypic information used to screen for hub genes associated with phenotypes (20).

#### 2.5.5 Screening of the critical genes

The candidate critical genes were identified as those genes that were identical to DEGs and hub genes. Subsequently, the receiver operating characteristic (ROC) curve model was verified using the pROC R package for the selected critical genes (20).

#### 2.5.6 Quantitative real-time polymerase chain reaction validation

Gene expression levels were assessed through the utilization of quantitative real-time polymerase chain reaction (qRT-PCR). The Reverse Transcription Kit was used to reverse-transcribe total RNA into cDNA (R222, Vazyme, Nanjing, China). The qRT-PCR was carried out using the SYBR Green master mix (Q311, Vazyme, Nanjing, China). Real-time detection of SYBR Green fluorescence was performed using a qTOWER 2.2 Real-Time PCR System (Analytik Jena, Jena, Germany). The *GAPDH* gene was amplified as an internal control. The relative quantification values for the critical genes were determined using the 2^−ΔΔCt^ method (21). Gene-specific primers were designed using Primer-BLAST^3^ and were listed in Supplementary Table S4.

#### 2.5.7 Functional enrichment analysis

The ClusterProfiler R package was employed to conduct Gene Ontology (GO) enrichment analysis of the DEGs. The GSEA R package was utilized to perform Gene Set Enrichment Analysis (GSEA) on the GO enrichment dataset. GO enrichments with a false discovery rate (FDR) less than 0.05 were considered statistically significant. GSEA enrichments with *p*-values less than 0.01 were considered significant (20, 22).

### 2.6 Metabolite extraction and untargeted metabolomic analysis

A total of 28 liver samples (7 sheep per group) were subjected to metabolite extraction using standard procedures (23). The extracted samples were then analyzed using a Vanquish UHPLC system coupled with an Orbitrap Q ExactiveTM HF mass spectrometer (ThermoFisher, MA, United States). The chromatographic and mass spectrometric conditions are shown in Supplementary Table S5.

The raw data files obtained from UHPLC–MS/MS analysis were processed using Compound Discoverer 3.1 (CD3.1, ThermoFisher) software. This involved peak alignment, peak picking, and quantification for each metabolite. Subsequently, the peak intensities were normalized to the total spectral intensity. The normalized data were used to predict the molecular formula based on additive ions, molecular ion peaks, and fragment ions. And then peaks were matched with the mzCloud, mzVault, and MassList databases to obtain accurate qualitative and relative quantitative results.

Differential metabolites (DEMs) between LSe and LCG, as well as SSe and SCG, were identified based on the following criteria: variable importance in projection (VIP) ≥ 1, |log2 fold change| ≥ 1, and *p* < 0.05. The enriched pathways associated with these DEMs were further investigated using the Kyoto Encyclopedia of Genes and Genomes (KEGG) database.^4^ The functions of the enriched pathways associated with the differential metabolites (DEMs) were investigated using the Kyoto Encyclopedia of Genes and Genomes (KEGG) database (see text footnote 4). The metabolic pathways with a *p*-value less than 0.05 were considered to exhibit statistically significant enrichment (24, 25).

### 2.7 Statistical analysis

The normality of the data distribution was assessed using the Shapiro–Wilk test. The independent samples *t*-test was performed to determine the significant difference between liver weight, liver mineral element concentration, and gene expression between the control group and treatment group using GraphPad Prism (v.9.3.1). The results were presented as mean ± standard error (SE), and differences with a *p*-value less than 0.05 were considered statistically significant. Pearson’s correlation analysis was conducted between critical genes and DEMs using GraphPad Prism (version 9.3.1). Correlations were considered significant if the *p*-value was less than 0.05 and the absolute value of the correlation coefficient (*r*) was greater than 0.7 (21). The results were visualized using R (version 4.1.2), and vector drawing was performed using Adobe Illustrator 2020.

## 3 Results

### 3.1 Liver fresh weight and mineral element concentration

**Figure 2:**
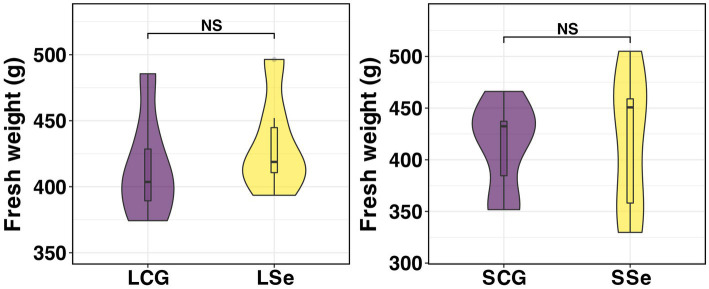
Fresh weight of grazing sheep liver. ^NS^ *p* > 0.05, * *p* < 0.05, ** *p* < 0.01, *** *p* < 0.001. LSe, the selenium deficient group; LCG, the control group of the selenium deficient treatment period; SSe, the selenium supplement group; SCG, the control group of the selenium supplement treatment period.

As depicted in Figure 2, dietary Se deficiency and supplementation had no impact on the liver weight of Wu Ranke sheep.

**Figure 3:**
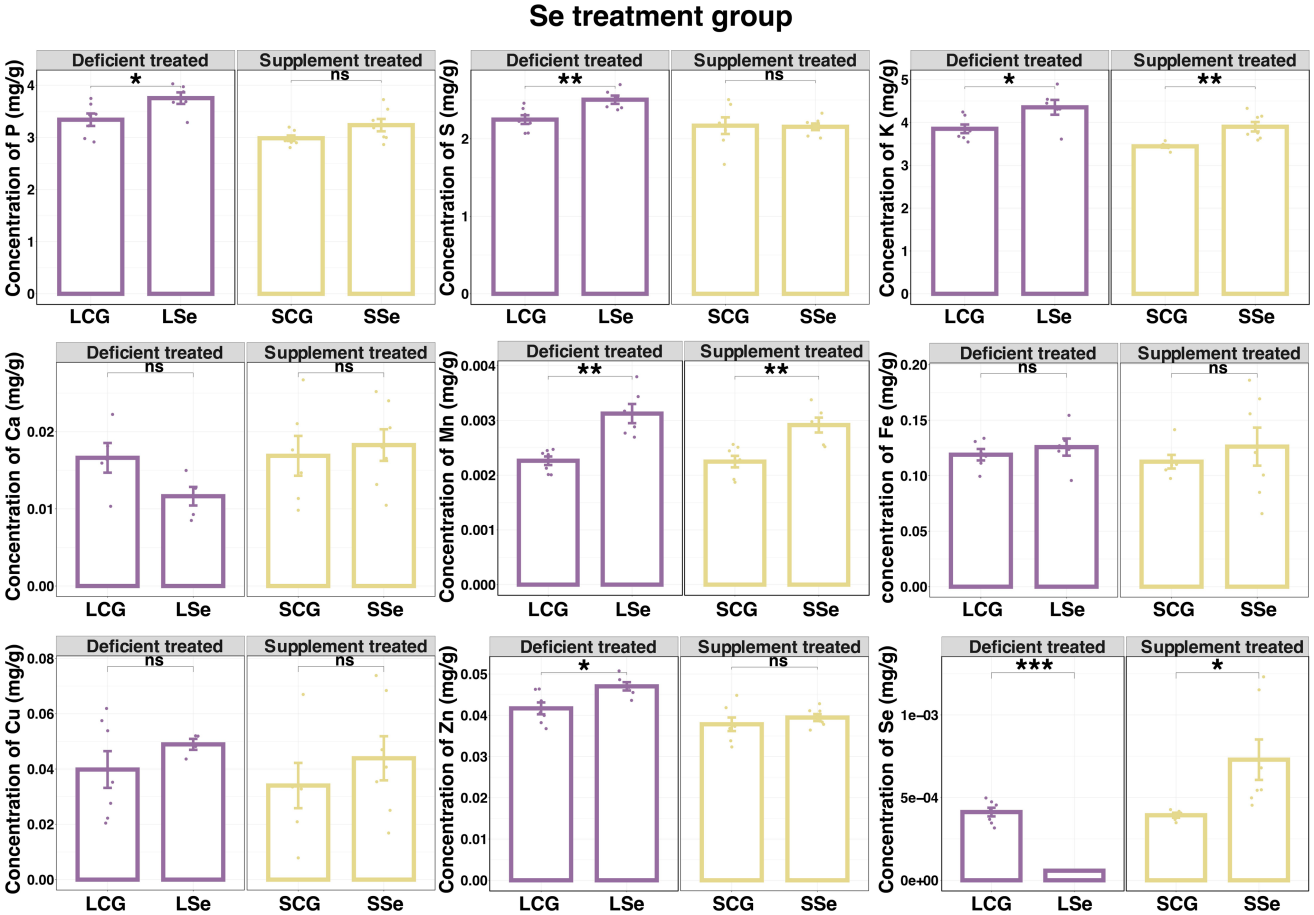
The concentration of liver mineral elements in selenium deficient treated and supplement treated compared with the control group. ^NS^ *p* > 0.05, * *p* < 0.05, ** *p* < 0.01, *** *p* < 0.001.

Figure 3 illustrates the liver concentration of 10 mineral elements in the Se deficient and supplement treatments.

Dietary Se deficient treatment caused a sharp decrease in the liver concentration of Se (0.000058 ± 0.0000011 mg/g vs. 0.00041 ± 0.000026 mg/g, *p* < 0.05), while significantly elevating the liver concentration of P (3.75 ± 0.11 mg/g vs. 3.34 ± 0.12 mg/g, *p* < 0.05), S (2.50 ± 0.053 mg/g vs. 2.25 ± 0.056 mg/g, *p* < 0.05), K (4.35 ± 0.17 mg/g vs. 3.85 ± 0.10 mg/g, *p* < 0.05), Mn (0.0031 ± 0.00018 mg/g vs. 0.0023 ± 0.000078 mg/g, *p* < 0.05) and Zn (0.047 ± 0.0010 mg/g vs. 0.042 ± 0.0014 mg/g, *p* < 0.05). This treatment did not affect the liver concentration of Ca, Fe, Cu, and Co was undetectable below the lower limit of instrumental detection.

Dietary Se supplement significantly elevated liver concentration of K (3.90 ± 0.11 mg/g vs. 3.44 ± 0.031 mg/g, *p* < 0.05), Mn (0.0029 ± 0.00014 mg/g vs. 0.0022 ± 0.00011 mg/g, *p* < 0.05), and Se (0.00073 ± 0.00012 mg/g vs. 0.00039 ± 0.000015 mg/g, *p* < 0.05), without affecting the concentration of P, S, Ca, Fe, Cu, Zn, and Co was undetectable below the instrumental detection limit.

### 3.2 Transcriptome analysis

#### 3.2.1 Identification of DEGs

**Figure 4:**
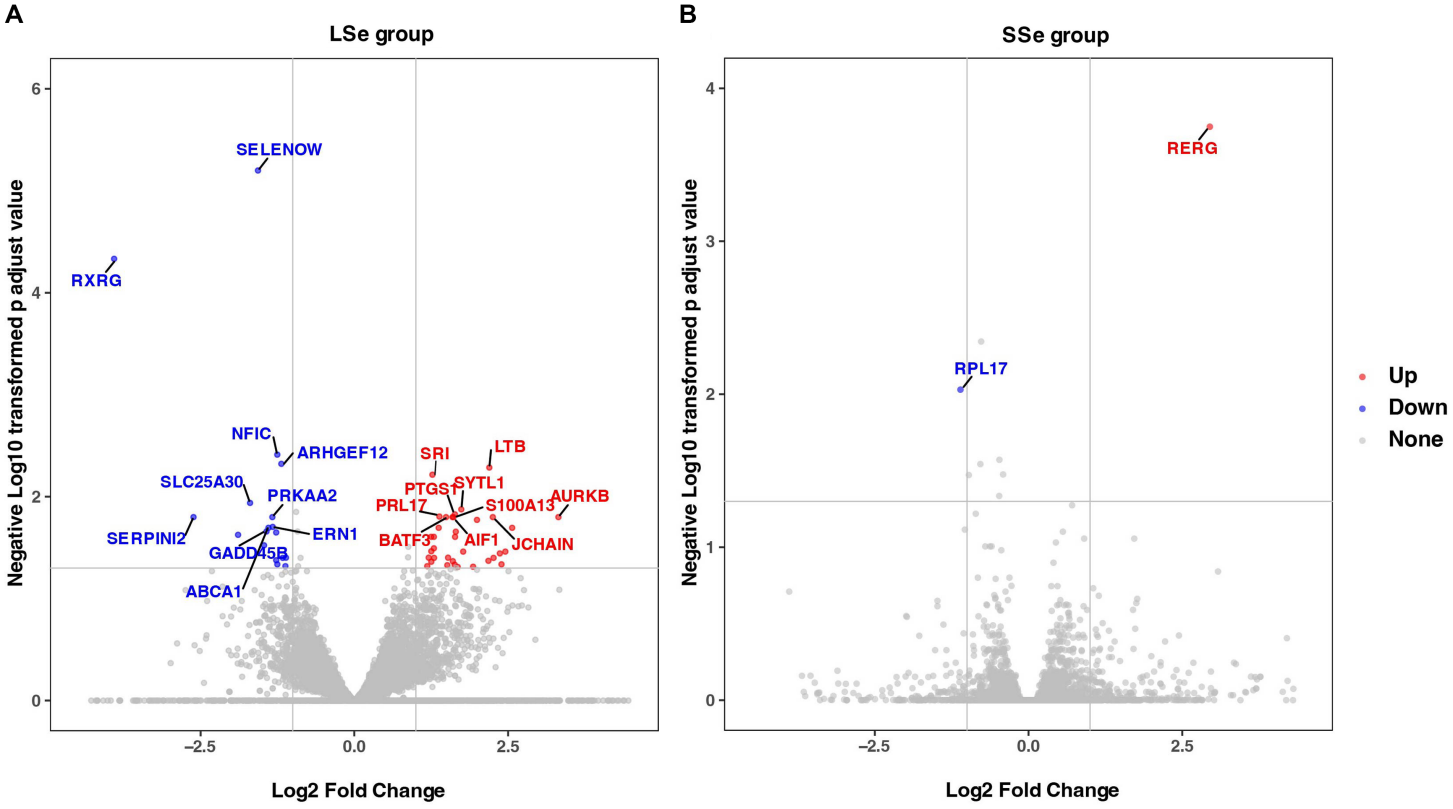
DEGs identified between different treatment groups. **(A)** Volcano Plot of DEGs in the selenium deficient group. **(B)** Volcano Plot of DEGs in the selenium supplement group. The top 10 up-regulated and down-regulated gene names were tagged and sorted by FDR value. DEGs, differentially expressed genes; FDR, false discovery rate.

Supplementary Figure S1 demonstrated that the sequence quality and per sequence quality scores of LSe, SSe, LCG, and SCG reflect the high quality of the RNA-seq data. To identify DEGs, the gene expression data was normalized using DESeq2. The normalized data can be observed in Supplementary Figure S2. Differential analysis revealed that a total of 53 DEGs were identified in LSe compared to LCG, of which 35 were up-regulated and 18 were down-regulated (Figure 4A). A total of 1 up-regulated DEGs and 1 down-regulated DEGs were identified in SSe compared to SCG (Figure 4B). The information regarding the up-regulated and down-regulated DEGs can be found in Supplementary Table S6.

#### 3.2.2 Function enrichment analysis of DEGs

**Figure 5:**
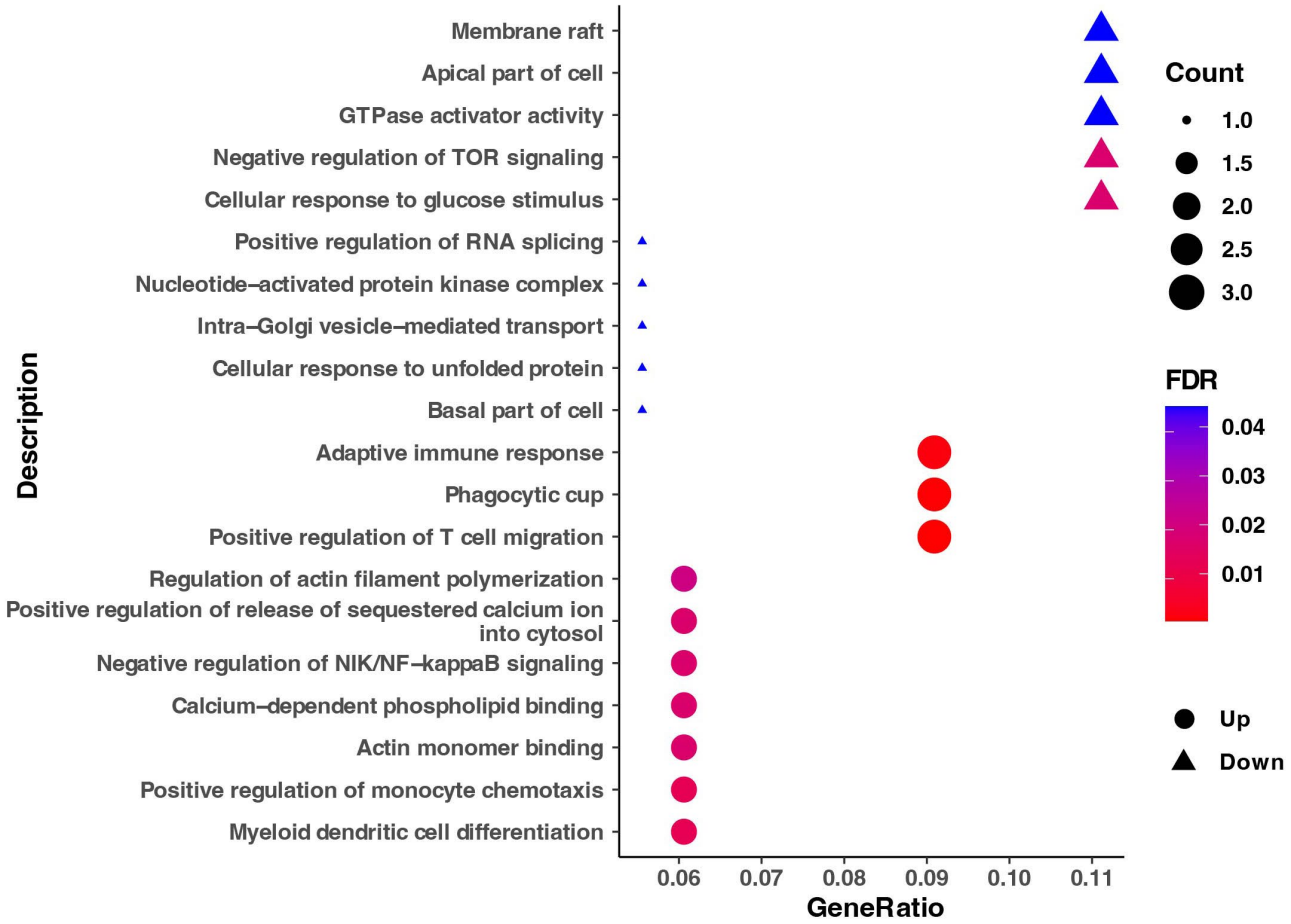
The Go enrichment of DEGs in the selenium supplement group. Go, gene ontology; DEGs, differentially expressed genes; FDR, false discovery rate.

As shown in Figure 5, the Go enrichment analysis revealed that the DEGs in LSe Wu Ranke sheep were significantly enriched in 20 terms compared with LCG, including cellular response to glucose stimulus, negative regulation of TOR signaling, apical part of cell, GTPase activator activity, and so on.

The Go enrichment analysis of DEGs in SSe Wu Ranke sheep indicated that DEGs were primarily significantly enriched in 6 terms, with down-regulated genes enriched in large ribosomal subunit, ribosome, structural constituent of ribosome, translation, and up-regulated genes enriched in GTPase activity and GTP binding.

**Figure 6:**
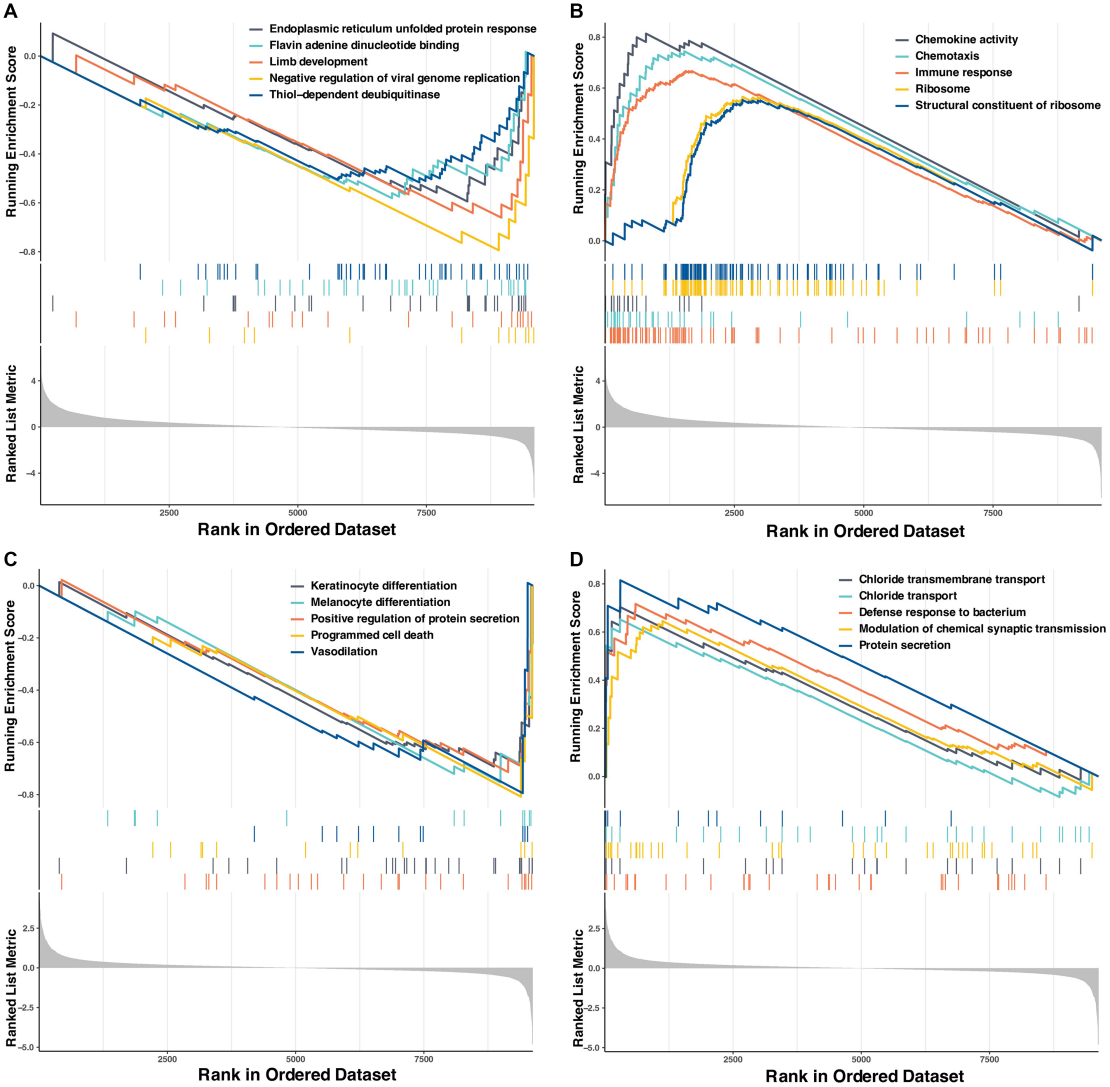
The Top 5 GSEA enrichment of Go terms. **(A)** Enriched in the control group of the selenium deficient treatment period. **(B)** Enriched in the selenium deficient group. **(C)** Enriched in the control group of the selenium supplement treatment period. **(D)** Enriched in the selenium supplement group. GSEA, gene set enrichment analysis; Go, gene ontology.

The results of GESA assays based on the Go enrichment data set are shown in Figure 6. The top 5 significantly enriched terms sorted by normalized enrichment score (NES) in LCG were thiol-dependent deubiquitinase (NES, −1.83), flavin adenine dinucleotide binding (NES, −1.83), endoplasmic reticulum unfolded protein response (NES, −1.84), limb development (NES, −1.92) and negative regulation of viral genome replication (NES, −2.03) (*p* < 0.01). The top 5 significantly enriched terms in LSe were immune response (NES, 2.28), chemotaxis (NES, 2.17), chemokine activity (NES, 2.08), ribosome (NES, 2.07), and structural constituent of ribosome (NES, 2.04) (*p* < 0.01). The top 5 significantly enriched terms in SCG were melanocyte differentiation (NES, −1.77), vasodilation (NES, −1.80), programmed cell death (NES, −1.86), keratinocyte differentiation (NES, −1.92) and positive regulation of protein secretion (NES, −1.95) (*p* < 0.01). The top 5 significantly enriched terms in SSe were defense response to the bacterium (NES, 1.83), chloride transmembrane transport (NES, 1.82), modulation of chemical synaptic transmission (NES, 1.80), chloride transport (NES, 1.75), and protein secretion (NES, 1.74) (*p* < 0.01).

#### 3.2.3 Screening of critical genes

**Figure 7:**
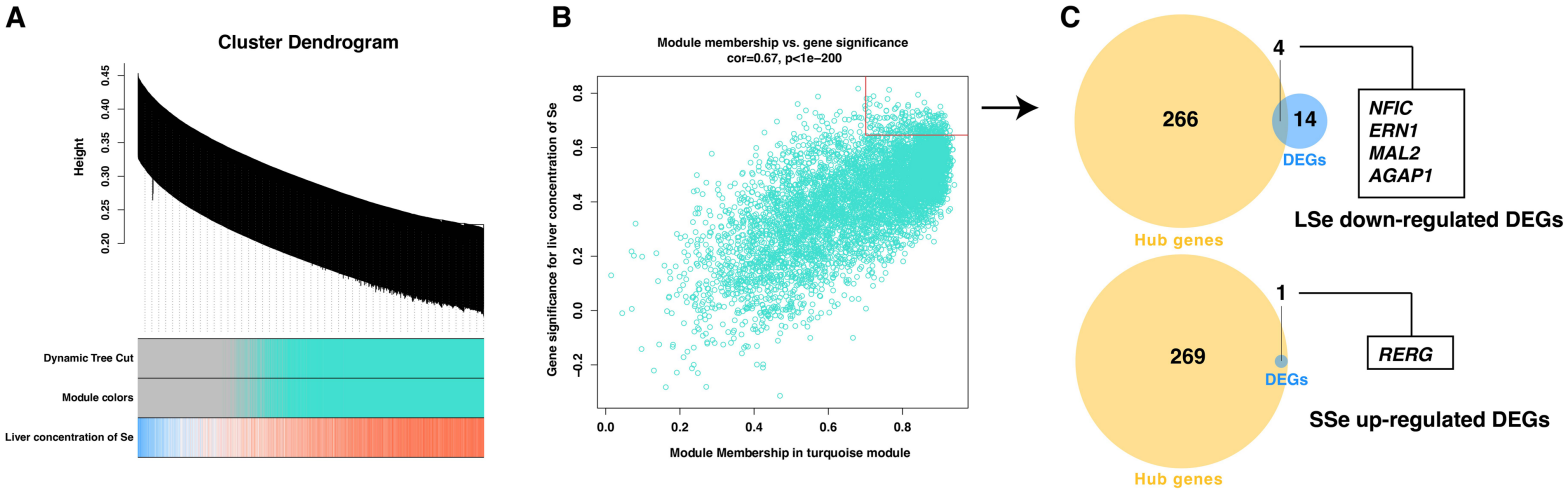
Screening of the candidate critical genes by WGCNA method. **(A)** Network dendrogram from co-expression topological overlap. Color bars show the correlation of gene expression with liver concentration of selenium. **(B)** The scatter plot of the association between the turquoise module and gene importance. The genes within the red box are identified as hub genes. **(C)** The Venn diagram of overlapping genes between DEGs and the hub genes as candidate critical genes. WGCNA, the weighted gene co-expression network analysis; DEGs, differentially expressed genes; LSe, the selenium deficient group; SSe, the selenium supplement group.

The WGCNA analysis resulted in the construction of a turquoise module. Therefore, the correlation data between the liver concentration of Se and the turquoise module were utilized to screen hub genes (Figure 7A). Hub genes of SSe up-regulated DEGs and LSe down-regulated DEGs were identified based on genes with module membership (MM) greater than 0.70 and gene significance (GS) greater than 0.64. A total of 270 hub genes were successfully screened (Figure 7B). The same method was employed to screen for hub genes among SSe down-regulated DEGs and LSe up-regulated DEGs. However, no hub genes were identified through this process.

Candidate critical genes were identified as the genes that overlapped between the DEGs and the hub genes. Ultimately, the 4 candidate hub genes were screened among the LSe down-regulated genes, and 1 candidate hub gene was screened among the SSe up-regulated genes (Figure 7C).

**Figure 8:**
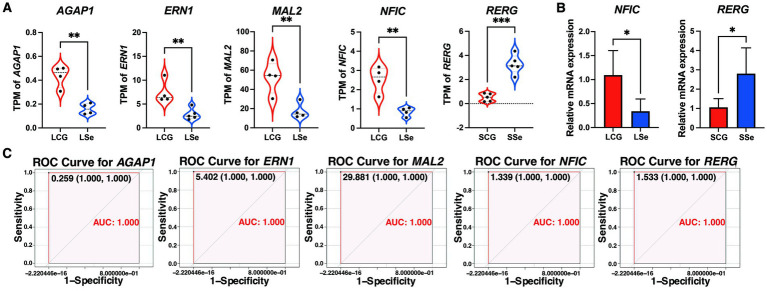
The validation of the critical genes. **(A)** The gene TPM counts of candidate critical genes. **(B)** The relative mRNA expression levels of *NFIC* and *RERG* in the liver by qRT-PCR. **(C)** The ROC validation for critical genes. ^NS^ *p* > 0.05, * *p* < 0.05, ** *p* < 0.01, *** *p* < 0.001. LSe, the selenium deficient group; LCG, the control group of the selenium deficient treatment period; SSe, the selenium supplement group; SCG, the control group of the selenium supplement treatment period; TPM, transcripts per kilobase million reads; qRT-PCR, quantitative real-time polymerase chain reaction; ROC, the receiver operating characteristic curve.

The gene expression of candidate critical genes in LSe and SSe is shown in Figure 8A. Two candidate genes, nuclear factor I C (*NFIC*) and RAS like estrogen regulated growth inhibitor (*RERG*), were randomly selected for qRT-PCR gene expression validation. As depicted in Figure 8B, the results demonstrated that the mRNA expression level of *NFIC* was significantly decreased and *RERG* was significantly increased compared with the control group (*p* < 0.05). The Figure 8C showed that the area under curve (AUC) values of the ROC models for ArfGAP with GTPase domain, ankyrin repeat and PH domain 1 (*AGAP1*), endoplasmic reticulum to nucleus signaling 1 (*ERN1*), mal, T cell differentiation protein 2 (*MAL2*), *NFIC* and *RERG* were 1, which indicated that *RERG* can be considered as critical gene for SSe and *AGAP1*, *ERN1*, *MAL2*, and *NFIC* can be considered as critical gene for LSe.

### 3.3 Metabolome analysis

#### 3.3.1 Identification of DEMs

**Figure 9:**
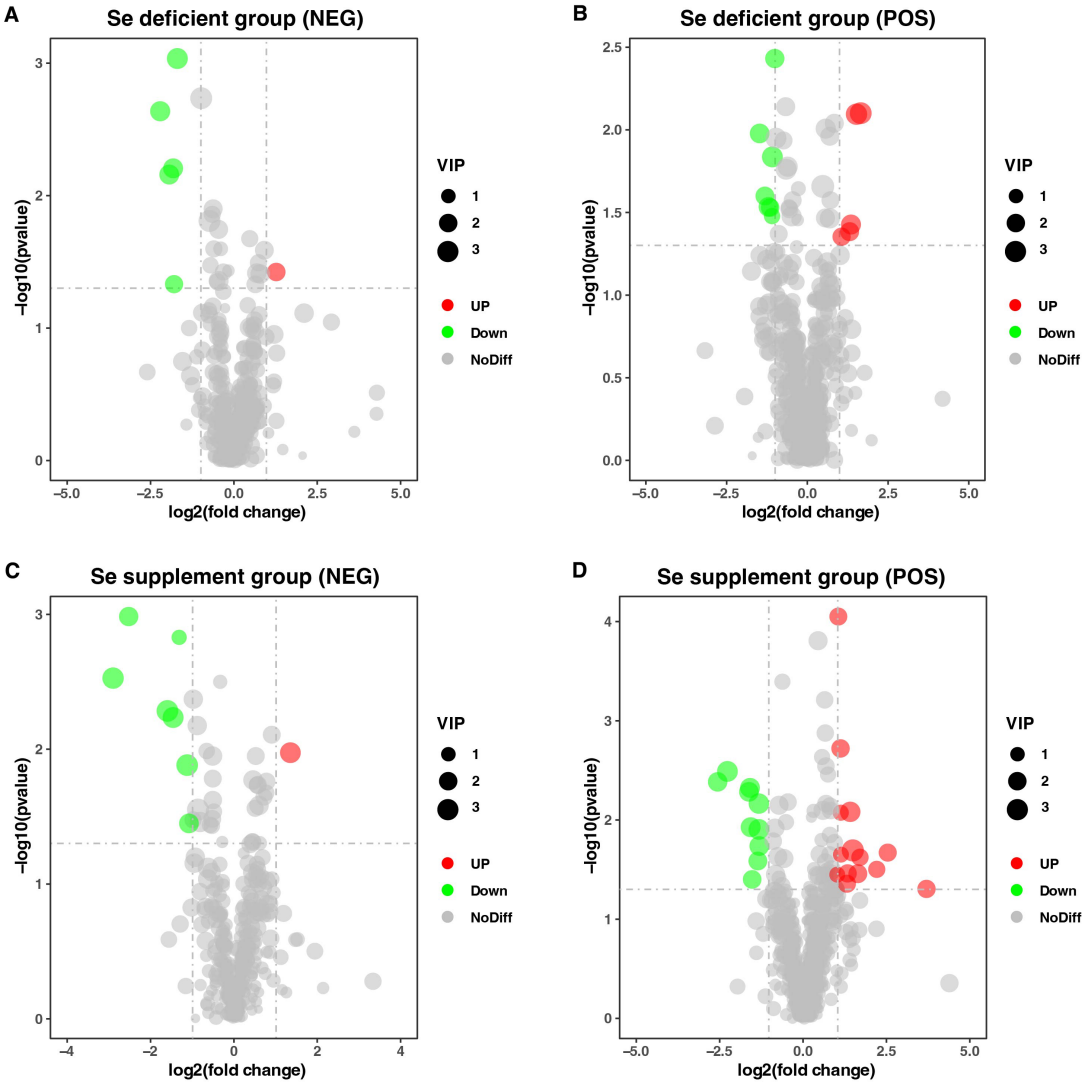
DEMs identified between different treatment groups. **(A)** Volcano Plot of DEMs in LSe of NEG. **(B)** Volcano Plot of DEMs in LSe of POS. **(C)** Volcano Plot of DEMs in SSe of NEG. **(D)** Volcano Plot of DEMs in SSe of POS. LSe, the selenium deficient group; SSe, the selenium supplement group; NEG, negative ion mode; POS, positive ion mode; DEMs, differential metabolites.

The Partial Least Squares Discriminant Analysis (PLS-DA) illustrated distinct differences between LSe and LCG, as well as between SSe and SCG, as depicted in Supplementary Figure S3. In Figures 9A,B, a total of 6 DEMs were identified in LSe using the negative ion mode (NEG), consisting of 1 up-regulated and 5 down-regulated DEMs (*p* < 0.05). Additionally, 12 DEMs were identified in LSe using the positive ion mode (POS), comprising 5 up-regulated and 7 down-regulated DEMs (*p* < 0.05). These DEMs were primarily classified into organooxygen compounds and carboxylic acids and derivatives. In the SSe (NEG), 8 DEMs were identified, including 1 up-regulated and 7 down-regulated DEMs, and 24 DEMs were identified in SSe (POS), including 14 up-regulated and 10 down-regulated DEMs, as shown in Figures 9C,D (*p* < 0.05). The DEMs of SSe were mainly classified into carboxylic acids and derivatives. The information regarding the up-regulated and down-regulated DEMs can be found in Supplementary Table S7.

#### 3.3.2 Function enrichment analysis of DEMs

**Figure 10:**
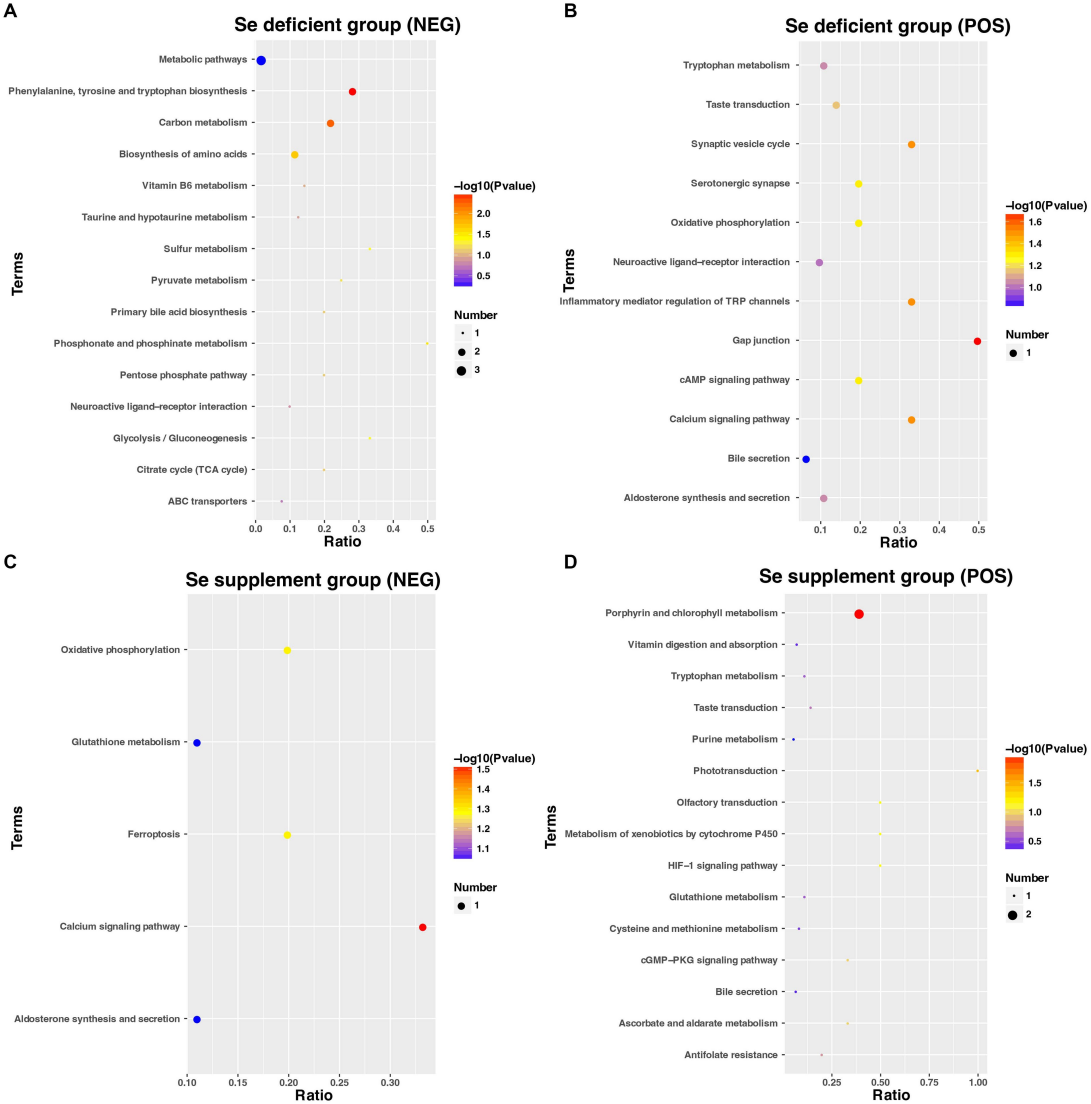
The KEGG pathways enrichment of DEMs in the LSe of NEG **(A)**, LSe of POS **(B)**, SSe of NEG **(C)**, and SSe of POS **(D)**. KEGG, the Kyoto Encyclopedia of Genes and Genomes; DEMs, differential metabolites; LSe, the selenium deficient group; SSe, the selenium supplement group; NEG, negative ion mode; POS, positive ion mode.

As depicted in Figure 10A, the results of the KEGG enrichment analysis revealed that the DEMs in LSe (NEG) were enriched in a total of 15 metabolic pathways. Among these pathways, Phenylalanine, tyrosine and tryptophan biosynthesis, Carbon metabolism, Biosynthesis of amino acids, Phosphonate and phosphinate metabolism, Glycolysis / Gluconeogenesis, and Sulfur metabolism exhibited significant enrichment (*p* < 0.05).

As shown in Figure 10B, the results of KEGG enrichment analysis indicated that the DEMs of LSe (POS) were enriched into a total of 12 metabolic pathways, of which Gap junction, Calcium signaling pathway, Synaptic vesicle cycle, and Inflammatory mediator regulation of TRP channels were significantly enriched (*p* < 0.05).

Figure 10C showed that the KEGG pathway demonstrated that the DEMs in SSe (NEG) were enriched in 5 pathways, of which the Calcium signaling pathway was significantly enriched (*p* < 0.05).

Figure 10D illustrated the KEGG pathway analysis, which revealed that the DEMs in SSe (POS) were enriched in 15 pathways. Among them, the Porphyrin and chlorophyll metabolism and Phototransduction exhibited significant enrichment among these pathways (*p* < 0.05).

The main secondary classifications of these KEGG pathways are Amino acid metabolism, Carbon metabolism, and Signal transduction.

### 3.4 Integrative analyses of transcriptome and metabolome

**Figure 11:**
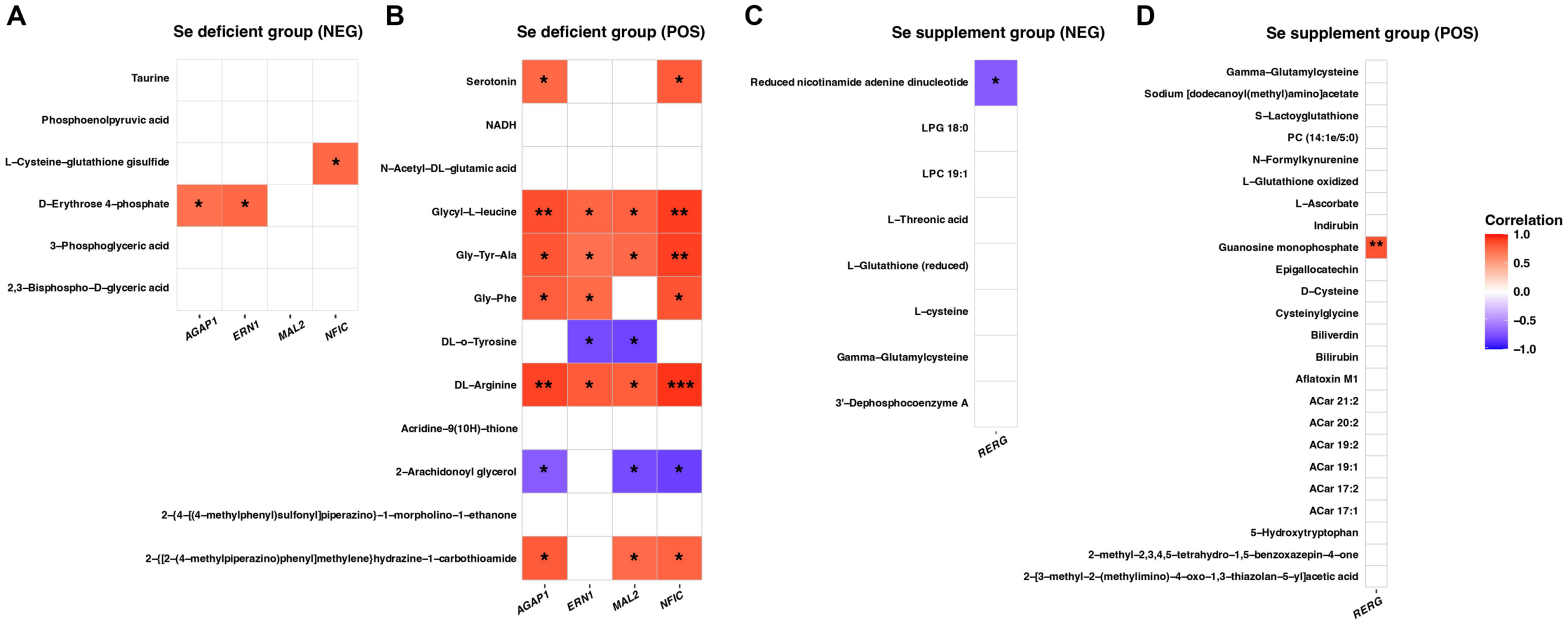
The correlation analysis of critical genes and DEMs in the LSe of NEG **(A)**, LSe of POS **(B)**, SSe of NEG **(C)**, and SSe of POS **(D)**. * *p* < 0.05, ** *p* < 0.01, *** *p* < 0.001. DEMs, differential metabolites; LSe, the selenium deficient group; SSe, the selenium supplement group; NEG, negative ion mode; POS, positive ion mode.

Correlation analysis between LSe critical genes and LSe DEMs (NEG) revealed that *AGAP1* and *ERN1* were significantly and positively correlated with D-Erythrose 4-phosphate, *NFIC* was significantly and positively correlated with L-Cysteine-glutathione disulfide (*p* < 0.05) (Figure 11A). In the LSe (POS), *AGAP1*, *ERN1*, *MAL2*, and *NFIC* were significantly and positively correlated with Gly-Tyr-Ala, Glycyl-L-leucine, and DL-Arginine. *AGAP1* and *NFIC* were significantly and positively correlated with Serotonin and Gly-Phe, while significantly and negatively correlated with 2-Arachidonoyl glycerol. *ERN1* was significantly and positively correlated with Gly-Phe, while significantly and negatively correlated with DL-o-Tyrosine. *MAL2* was significantly and negatively correlated with DL-o-Tyrosine and 2-Arachidonoyl glycerol (*p* < 0.05) (Figure 11B).

Correlation analysis between SSe critical gene and SSe DEMs indicated that *RERG* exhibited a significant negative correlation with Reduced nicotinamide adenine dinucleotide in the NEG and a significant positive correlation with Guanosine monophosphate in the POS (*p* < 0.05) (Figures 11C,D).

## 4 Discussion

Mineral elements play a vital role in the physiological functions and healthy development of sheep. Se, in particular, has been found to be crucial for the immune system, reproductive health, and antioxidant capacity of sheep (9). While numerous studies have explored the addition of organic or inorganic Se to sheep diets, comprehensive investigations into the specific effects of Se deficiency or supplementation on the liver of grazing sheep are lacking (10, 11, 26, 27). Therefore, this experiment delves into assessing liver weight, the concentration of mineral elements, transcriptomics, and metabolomics in grazing sheep under conditions of dietary Se deficiency and Se supplementation.

The results showed that dietary Se treatment had no significant impact on liver weights in grazing sheep. This suggested that changes in liver weight may not be directly regulated by the dietary Se status. Previous studies have demonstrated that the liver was the primary storage for Se in animals (10). The mineral elements results revealed a significant decrease in the liver concentration of Se in response to dietary Se deficiency. Conversely, dietary Se supplementation led to a significant increase in the liver concentration of Se. The findings of our experiment on liver mineral elements are consistent with previous studies, supporting that liver Se levels can assess the status of Se in grazing sheep. Furthermore, it was observed that dietary Se deficiency led to a significant increase in the liver concentration of S. This can be attributed to the similar chemical properties of S and Se, resulting in competition for the same transporter protein (28). Consequently, when the concentration of Se in the liver decreased, there was a significant increase in the concentration of S. Meanwhile, the dietary supplement Se resulted in a significant increase in the liver concentration of K and Mn. This phenomenon is likely attributed to the essential role of Se as a component of numerous enzymes (9). The supplementation of Se to the diet enhanced the activity of these enzymes, thereby promoting the absorption of K and Mn by the liver.

Transcriptome analysis can unveil a series of genes that display differential expression in grazing sheep when subjected to dietary Se deficiency or supplementation. Go enrichment analysis of DEGs revealed that dietary Se deficiency reduced the cellular response to glucose stimulus and decreased the efficiency of intra-Golgi vesicle-mediated transport. The Golgi vesicle was an essential organelle responsible for protein synthesis, modification, and transport within the cell (29). When the efficiency of intra-Golgi vesicle-mediated transport was compromised, the process of protein synthesis was negatively impacted, subsequently affecting cell metabolism and normal function. Furthermore, our findings indicated that dietary supplementation with Se significantly increased GTPase activity and the abundance of GTP-binding proteins, leading to enhanced protein synthesis (30). Moreover, GSEA analysis also demonstrated that the terms associated with protein secretion were significantly enriched in SSe. Meanwhile, the term related to flavin adenine dinucleotide (FAD) was found to be significantly enriched in LCG. FAD was a crucial coenzyme that played a vital role in regulating enzyme activities, participating in energy metabolism, and providing antioxidant protection (31). The above results indicate that dietary Se deficiency can have negative effects on protein synthesis and reduce the antioxidant capacity of liver cells, whereas dietary Se supplementation has the potential to enhance the protein synthesis capacity of liver cells in Wu Ranke sheep.

By employing WGCNA and DESeq2 analyses, four down-regulated genes *AGAP1*, *ERN1*, *MAL2*, and *NFIC* were screened as the critical gene for LSe, while up-regulated gene *RERG* was screened as the critical gene for SSe. Among them, *AGAP1* was widely recognized as a gene involved in the regulation of membrane transport and protein transport. Additionally, it has been linked to psychiatric disorders such as autism and schizophrenia (32). The NFI family is a crucial family of transcription factors involved in adenoviral DNA replication, which consists of 4 genes, including *NFIA*, *NFIB*, *NFIC*, and *NFIX*, in most vertebrates. Research has demonstrated that up-regulation of *NFIC* promotes adipocyte and osteoblast differentiation (33, 34). *RERG* was a tumor suppressor gene. In mice, the RERG protein has been shown to effectively inhibit tumorigenesis (35). It plays a critical role in regulating cell growth, differentiation, and survival. Moreover, *RERG* could be considered as a potential candidate gene associated with the promotion of goat breeding (36). These 5 genes can serve as valuable biomarkers for evaluating the Se status in grazing sheep. Furthermore, the findings suggest that dietary Se deficiency may have adverse effects on the differentiation of adipocytes and osteoblasts, as well as protein transport, while dietary Se supplementation may suppress tumorigenesis in Wu Ranke sheep.

Metabolomics analysis revealed that DEMs in LSe and SSe Wu Ranke sheep were predominantly classified as Organooxygen compounds and Carboxylic acids and derivatives. KEGG pathway enrichment analysis indicated that the DEMs were primarily enriched in metabolic pathways such as amino acid metabolism, carbohydrate metabolism, and signal transduction. This suggests that dietary Se deficiency and supplementation may affect amino acid metabolism and carbohydrate metabolism in the liver of Wu Ranke sheep.

The abundance of several metabolites, including L-Cysteine-glutathione disulfide (L-CySSG), Phosphoenolpyruvic acid (PEP), Serotonin, and DL-arginine, were significantly down-regulated in the liver DEMs of LSe Wu Ranke sheep. Among them, L-CySSG served as a storage form of L-cysteine and has been found to possess potent hepatoprotective properties against liver damage (37). PEP was a glycolytic and carbohydrate metabolite with a high-energy phosphoryl group, known for its cytoprotective and antioxidant activities (38). Serotonin, also known as 5-hydroxytryptamine, was a major product of tryptophan metabolism and can be produced through the decarboxylation of 5-hydroxytryptophan in the catalyze of tryptophan carboxylase. It has been shown that serotonin promotes liver regeneration following partial resection of the liver in animals (39, 40). Additionally, DL-arginine has been shown to inhibit lipid peroxidation and suppress inflammatory responses (41). The results of the integrative analysis of transcriptomics and metabolomics indicated that the down-regulated genes *NFIC*, *ERN1*, *MAL2*, and *AGAP1* were able to positively regulate the production of amino acid metabolites, specifically Gly-Tyr-Ala, Glycyl-L-leucine, and DL-arginine. Furthermore, *NFIC* was found to positively regulate the production of L-CySSG and serotonin, while *AGAP1* was able to positively regulate serotonin production. In light of these findings, the down-regulation of the above metabolites can be inferred that Se deficiency in the diet may lower the hepatic antioxidant capacity and anti-inflammatory response, rendering it susceptible to damage in Wu Ranke sheep. However, the specific functions and regulatory mechanisms of these observations will require further investigation.

The integrative analysis of transcriptomics and metabolomics of SSe indicated that the up-regulated gene *RERG* was able to positively regulate the production of guanosine monophosphate (GMP), while negatively regulating the production of reduced nicotinamide adenine dinucleotide (NADH). Among them, GMP was a nucleotide that served as a fundamental building block of RNA and DNA. It played a crucial role in various physiological processes, including protein synthesis and energy metabolism (42). NADH acted as an electron transfer mediator in redox reactions in animals, which facilitated the production of mitochondrial ATP in the cellular respiratory chain (43). The metabolomics result also revealed that the abundance of several metabolites, including L-glutathione oxidized (GSSG), epigallocatechin (EGC), and 5-hydroxytryptophan, were significantly up-regulated in the liver DEMs of SSe Wu Ranke sheep. Among them, GSSG was a tripeptide composed of glycine, cysteine, and glutamic acid. It played a crucial role in cellular antioxidant defense by converting between its oxidized and reduced forms through the catalytic action of glutathione reductase. The oxidation of glutathione to GSSG was an essential mechanism for cells to balance reactive oxygen species and reduce oxidative stress. This was particularly important as free radicals have the ability to react with intracellular molecules, causing cell damage and triggering apoptosis. By reacting with free radicals, GSSG helps protect cells from oxidative damage by reducing the number of free radicals (44). EGC, a tea polyphenol, has been found to have significant preventive effects against cardiovascular disease and cancer in animal studies (45). Moreover, when combined with curcumin, EGC exhibited inhibitory effects on inflammation in animals (46). Additionally, 5-hydroxytryptophan served both as a drug and a dietary supplement. It can be decarboxylated to produce 5-hydroxytryptamine, which is further converted into melatonin. As a monoamine neurotransmitter, it regulates various physiological processes including mood, cognition, learning, memory, and sleep. Furthermore, it possessed anti-inflammatory properties by reducing the production of pro-inflammatory mediators. Studies conducted on the liver of Holstein cows have shown that 5-hydroxytryptophan stimulated autocrine-paracrine adaptation to lactation. Interestingly, dietary Se deficiency significantly decreased the abundance of 5-hydroxytryptamine in the liver metabolites of Wu Ranke sheep. Conversely, dietary Se supplementation led to a significant increase in the abundance of 5-hydroxytryptophan in the liver metabolites of Wu Ranke sheep. This suggested that dietary Se supplementation may enhance the antioxidant, anti-inflammatory, and lactation capacities of Wu Ranke sheep. However, further analysis is required to elucidate the specific mechanisms involved.

In addition, there are several limitations in this study that should be acknowledged. Firstly, the feeding experiment could have been designed optimally for 30, 60, and 90 days to obtain more reasonable days for supplemental feeding. Secondly, the dosage of supplemental Se could be set to different concentration gradients to explore the optimal supplemental dose of grazing. In future experiments, we will further investigate the mechanisms through which critical genes influence the abundance of metabolites. Additionally, we also intend to delve deeper into the specific mechanisms by which hepatic critical genes regulate Se deficiency and supplementation in grazing sheep. Furthermore, we have plans for further research involving diverse grazing sheep breeds to validate and expand upon our findings, ultimately enhancing the depth and breadth of our research insights.

## 5 Conclusion

This study aimed to investigate the effect of dietary Se deficiency and supplementation on the liver weight, the concentration of mineral elements, transcriptomic, and metabolomic in grazing Wu Ranke sheep. The findings revealed that the concentration of Se in the liver was found to be susceptible to variations in dietary Se levels. Go and GESA enrichment analysis suggested that dietary Se deficiency might impair protein synthesis efficiency, while Se supplementation was found to be beneficial in enhancing liver protein synthesis in grazing sheep. *AGAP1*, *ERN1*, *MAL2*, *NFIC*, and *RERG* were identified as critical genes through WGCNA, qRT-PCR and ROC validation that could potentially serve as biomarkers. Integrative analysis of the transcriptome and metabolome revealed that dietary Se deficiency led to reduced hepatic antioxidant capacity and anti-inflammatory response, whereas Se supplementation increased the hepatic antioxidant and anti-inflammatory capacity in grazing Wu Ranke sheep.

## Data availability statement

The datasets presented in this study can be found in online repositories. The names of the repository/repositories and accession number(s) can be found at: https://www.ncbi.nlm.nih.gov/, PRJNA1031778.

## Ethics statement

The animal study was approved by Inner Mongolia University Animal Care and Use Committee. The study was conducted in accordance with the local legislation and institutional requirements.

## Author contributions

XJ: Data curation, Software, Writing – original draft. LiM: Software, Validation, Visualization, Writing – original draft. ZQ: Conceptualization, Resources, Methodology, Writing – review & editing. LaM: Writing – review & editing.

## Footnotes

^1^https://www.bioinformatics.babraham.ac.uk/projects/fastqc

^2^http://www.ensembl.org/index.html

^3^https://www.ncbi.nlm.nih.gov/tools/primer-blast/

^4^https://www.genome.jp/kegg/pathway.html
