# Supplementary material for: Effects of dietary selenium deficiency and supplementation on liver in grazing sheep: insights from transcriptomic and metabolomic analysis
Source: Front Vet Sci. 2024 Jun 19;11:1358975. doi: 10.3389/fvets.2024.1358975 (PMC11220315; doi:10.3389/fvets.2024.1358975)
Supplement: Supplementary file 1 [file Data_Sheet_1.docx]

**Effects of dietary selenium deficiency and supplementation on liver in grazing sheep: insights from transcriptomic and metabolomic analysis**

Xiwei Jin, Lingbo Meng, Zhi Qi^*^ and Lan Mi^*^

**Supplementary material**

**Fig. S1.** The result of FastQC quality control. The sequence quality of LSe and LCG (A), SSe and SCG (C). The per sequence quality scores of LSe and LCG (B), SSe and SCG (D). Abbreviations: LSe = the selenium deficient group; LCG = the control group of the selenium deficient treatment period; SSe = the selenium supplement group; SCG = the control group of the selenium supplement treatment period.


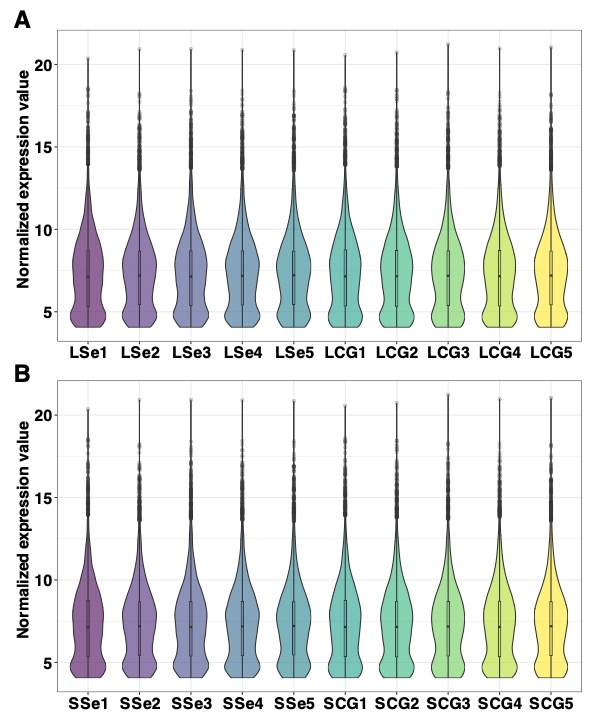


**Fig. S2.** The normalized expression value of LSe and LCG (A), SSe and SCG (B) by DESeq2. Abbreviations: LSe = the selenium deficient group; LCG = the control group of the selenium deficient treatment period; SSe = the selenium supplement group; SCG = the control group of the selenium supplement treatment period.

**Fig. S3.** The partial least squares discriminant analysis of LSe and LCG (A, B), SSe and SCG (C, D). R2Y values higher than Q2Y values indicate that the model quality is reliable. Abbreviations: LSe = the selenium deficient group; LCG = the control group of the selenium deficient treatment period; SSe = the selenium supplement group; SCG = the control group of the selenium supplement treatment period; NEG = negative ion mode; POS = positive ion mode.

**Table S1 Formulation of the multi-nutrient salts for grazing Wu Ranke sheep**

| **Group** | **Types** | **Amounts** | **Types** | **Amounts** | **Types** | **Amounts** |
| --- | --- | --- | --- | --- | --- | --- |
| **Control group** | KCl | 11.5 g/d | MgO | 1.7 g/d | Ca(IO_3_)_2_ | 1.4 mg/d |
|  | CaCO_3_ | 10 g/d | FeSO_4_ | 200 mg/d | CoSO_4_ | 1.2 mg/d |
|  | Na_2_SO_4_ | 9 g/d | ZnSO_4_ | 132 mg/d | Na_2_SeO_3_ | 1 mg/d |
|  | NH_4_H_2_PO_4_ | 7.4 g/d | MnSO_4_ | 92 mg/d |  |  |
|  | NaCl | 5 g/d | CuSO_4_ | 36 mg/d |  |  |
| **Se**  **deficient group** | KCl | 11.5 g/d | MgO | 1.7 g/d | Ca(IO_3_)_2_ | 1.4 mg/d |
|  | CaCO_3_ | 10 g/d | FeSO_4_ | 200 mg/d | CoSO_4_ | 1.2 mg/d |
|  | Na_2_SO_4_ | 9 g/d | ZnSO_4_ | 132 mg/d | Na_2_SeO_3_ | 0 mg/d |
|  | NH_4_H_2_PO_4_ | 7.4 g/d | MnSO_4_ | 92 mg/d |  |  |
|  | NaCl | 5 g/d | CuSO_4_ | 36 mg/d |  |  |
| **Se supplement group** | KCl | 11.5 g/d | MgO | 1.7 g/d | Ca(IO_3_)_2_ | 1.4 mg/d |
|  | CaCO_3_ | 10 g/d | FeSO_4_ | 200 mg/d | CoSO_4_ | 1.2 mg/d |
|  | Na_2_SO_4_ | 9 g/d | ZnSO_4_ | 132 mg/d | Na_2_SeO_3_ | 3 mg/d |
|  | NH_4_H_2_PO_4_ | 7.4 g/d | MnSO_4_ | 92 mg/d |  |  |
|  | NaCl | 5 g/d | CuSO_4_ | 36 mg/d |  |  |

**Table S2** **Nutrient content in oats and natural grass**

| **Items** | **Oats** | **Natural grass** |
| --- | --- | --- |
| P (mg/g) | 2.40 | 0.65 |
| S (mg/g) | 1.39 | 1.09 |
| K (mg/g) | 5.05 | 11.83 |
| Ca (mg/g) | 0.52 | 7.43 |
| Mn (μg/g) | 41.00 | 54.00 |
| Fe (μg/g) | 112.00 | 566.00 |
| Co (μg/g) | - | - |
| Cu (μg/g) | 3.50 | 4.50 |
| Zn (μg/g) | 21.00 | 17.00 |
| Se (μg/g) | 0.090 | - |
| CP (g/kg) | 103.65 | 82.5 |
| ADF (g/kg) | 52.71 | 301.51 |
| NDF (g/kg) | 117.16 | 650.68 |

**Table S3 The digestion procedure of the microwave digester**

| **steps** | **Control temperature (℃)** | **Heating time (min)** | **Constant temperature time (min)** |
| --- | --- | --- | --- |
| 1 | 120 | 5 | 5 |
| 2 | 150 | 5 | 10 |
| 3 | 190 | 5 | 20 |

**Table S4 The primers sequences**

| **Gene name** | **Forward (5’ to 3’)** | **Reserve (5’ to 3’)** |
| --- | --- | --- |
| *NFIC* | CAGTTCTCCCACGAGTAGCA | AGATGGGCTGTTGAAAGGAG |
| *RERG* | GTGCAGAAGTCAAACTGGCA | CAATGGTTGCTTGGTGTCGG |
| *GAPDH* | CGTGTCCGTTGTGGATCTGA | TGAAGTCGCAGGAGACAACC |

**Table S5 The chromatographic and** **mass spectrometric conditions of UHPLC-MS/MS**

|  | **Type** | **Parameters** |
| --- | --- | --- |
| **The**  **chromatographic**  **conditions** | Chromatographic column | Hypesil Goldcolumn (C18) |
|  | Column temperature | 40 °C |
|  | Flow rate | 0.2 mL/min |
|  | Positive polarity mode | Eluent A (0.1% FA in Water) |
|  |  | Eluent B (Methanol) |
|  | Negative polarity mode | Eluent A (5 mM ammonium acetate, pH 9.0) |
|  |  | Eluent B (Methanol) |
| **The**  **mass**  **spectrometric**  **conditions** | Spray Voltage | 3.2 kV |
|  | Sheath gas flow rate | 40 arb |
|  | Aux Gasflow rate | 10 arb |
|  | Capillary Temp | 320 °C |
|  | Polarity | Positive; negative |

**Table S6 The differentially expressed genes identified in Se treatment group*^a^***

| **Group** | **Genes** | **Log_2_FC** | **FDR** | **Up/Down** |
| --- | --- | --- | --- | --- |
| LSe | *SELENOW* | -1.567 | 6.34E-06 | Down |
|  | *RXRG* | -3.905 | 4.66E-05 | Down |
|  | *NFIC* | -1.253 | 0.003878 | Down |
|  | *ARHGEF12* | -1.187 | 0.004772 | Down |
|  | *LTB* | 2.196 | 0.005175 | Up |
|  | *SRI* | 1.27 | 0.006109 | Up |
|  | *SLC25A30* | -1.696 | 0.0115 | Down |
|  | *SYTL1* | 1.739 | 0.01334 | Up |
|  | *PTGS1* | 1.635 | 0.01491 | Up |
|  | *RPL17* | 1.383 | 0.01568 | Up |
|  | *PRKAA2* | -1.333 | 0.01585 | Down |
|  | *JCHAIN* | 2.251 | 0.01585 | Up |
|  | *BATF3* | 1.492 | 0.01585 | Up |
|  | *SERPINI2* | -2.615 | 0.01585 | Down |
|  | *S100A13* | 1.612 | 0.01585 | Up |
|  | *AIF1* | 1.598 | 0.01585 | Up |
|  | *AURKB* | 3.32 | 0.01585 | Up |
|  | *CD52* | 1.994 | 0.01693 | Up |
|  | *ERN1* | -1.328 | 0.01977 | Down |
|  | *ABCA1* | -1.397 | 0.02023 | Down |
|  | *CCL21* | 2.567 | 0.02023 | Up |
|  | *ANXA1* | 1.372 | 0.02023 | Up |
|  | *GADD45B* | -1.428 | 0.02192 | Down |
|  | *CXCL17* | 1.649 | 0.02211 | Up |
|  | *MAL2* | -1.27 | 0.02247 | Down |
|  | *H6PD* | -1.891 | 0.02369 | Down |
|  | *SLAMF7* | 1.638 | 0.02482 | Up |
|  | *ACP5* | 1.25 | 0.02482 | Up |
|  | *ARPC1B* | 1.293 | 0.02482 | Up |
|  | *NBEAL1* | -1.467 | 0.03 | Down |
|  | *SPI1* | 1.293 | 0.03208 | Up |
|  | *IER5L* | 1.252 | 0.03431 | Up |
|  | *SH2D1A* | 2.456 | 0.03458 | Up |
|  | *LGALS1* | 1.77 | 0.03458 | Up |
|  | *SLC44A4* | 2.367 | 0.03601 | Up |
|  | *SH3BGRL3* | 1.211 | 0.03963 | Up |
|  | *UBR2* | -1.11 | 0.03963 | Down |
|  | *DDAH2* | 1.524 | 0.03963 | Up |
|  | *RGS19* | 1.298 | 0.03977 | Up |
|  | *AP4E1* | -1.164 | 0.03977 | Down |
|  | *CXCL9* | 2.265 | 0.03977 | Up |
|  | *SLC11A2* | -1.269 | 0.04199 | Down |
|  | *CTNND2* | 2.181 | 0.04255 | Up |
|  | *CORO1A* | 1.602 | 0.04301 | Up |
|  | *ThymB4X* | 1.251 | 0.04345 | Up |
|  | *ADIRF* | 2.393 | 0.04601 | Up |
|  | *MPP5* | -1.252 | 0.04612 | Down |
|  | *ANXA13* | 1.633 | 0.04656 | Up |
|  | *LIMD2* | 1.512 | 0.04716 | Up |
|  | *RPL34* | 1.186 | 0.04802 | Up |
|  | *AGAP1* | -1.12 | 0.04802 | Down |
|  | *MLF1* | 1.933 | 0.04875 | Up |
|  | *COTL1* | 1.679 | 0.04878 | Up |
| SSe | *RERG* | 2.94 | 0.00018 | Up |
|  | *RPL17* | -1.107 | 0.00933 | Down |

*^a^*LSe: the selenium deficient group, SSe: the selenium supplement group, Log_2_FC: Log_2_Fold change.

**Table S7** **The differential metabolites identified in the Se treatment group*^a^***

| **Group** | **Metabolites** | **VIP** | **Log_2_FC** | ***p* value** | **Up/Down** |
| --- | --- | --- | --- | --- | --- |
| **LSe (NEG)** | D-Erythrose 4-phosphate | 2.21366 | -1.69498 | 0.00092 | Down |
|  | L-Cysteine-glutathione disulfide | 1.98338 | -2.20935 | 0.00231 | Down |
|  | Phosphoenolpyruvic acid | 1.91691 | -1.82023 | 0.00622 | Down |
|  | 3-Phosphoglyceric acid | 1.92587 | -1.93711 | 0.00694 | Down |
|  | Taurine | 1.58156 | 1.27126 | 0.03774 | Up |
|  | 2,3-Bisphospho-D-glyceric acid | 1.51827 | -1.79360 | 0.04662 | Down |
| **LSe (POS)** | Gly-Tyr-Ala | 1.77781 | -1.00750 | 0.00370 | down |
|  | acridine-9(10H)-thione | 2.47853 | 1.65440 | 0.00793 | up |
|  | N-Acetyl-DL-glutamic acid | 2.33484 | 1.52206 | 0.00804 | up |
|  | Glycyl-L-leucine | 1.87746 | -1.47637 | 0.01052 | down |
|  | DL-Arginine | 2.21379 | -1.08256 | 0.01458 | down |
|  | Gly-Phe | 1.66912 | -1.31423 | 0.02513 | down |
|  | 2-{[2-(4-methylpiperazino)phenyl]methylene}hydrazine-1-carbothioamide | 1.86255 | -1.20543 | 0.02925 | down |
|  | Serotonin | 1.58157 | -1.15028 | 0.02952 | down |
|  | 2-{4-[(4-methylphenyl)sulfonyl]piperazino}-1-morpholino-1-ethanone | 1.10747 | -1.09255 | 0.03325 | down |
|  | DL-o-Tyrosine | 1.95587 | 1.35275 | 0.03739 | up |
|  | 2-Arachidonoyl glycerol | 1.70499 | 1.31019 | 0.04131 | up |
|  | NADH | 1.52792 | 1.06106 | 0.04431 | up |
| **SSe (NEG)** | L-Glutathione (reduced) | 2.13120 | -2.52354 | 0.00104 | down |
|  | Reduced nicotinamide adenine dinucleotide | 1.08181 | -1.31259 | 0.00148 | down |
|  | 3'-Dephosphocoenzyme A | 2.78419 | -2.89846 | 0.00297 | down |
|  | gamma-Glutamylcysteine | 2.89025 | -1.59449 | 0.00520 | down |
|  | L-cysteine | 2.63158 | -1.45766 | 0.00582 | down |
|  | L-Threonic acid | 2.58413 | 1.35588 | 0.01061 | up |
|  | LPG 18:0 | 2.88108 | -1.12097 | 0.01312 | down |
|  | LPC 19:1 | 2.24535 | -1.07748 | 0.03550 | down |
| **SSe (POS)** | Guanosine monophosphate | 1.90332 | 1.05797 | 0.00009 | up |
|  | Sodium [dodecanoyl(methyl)amino]acetate | 2.05805 | 1.12342 | 0.00190 | up |
|  | S-Lactoyglutathione | 2.86055 | -2.26750 | 0.00324 | down |
|  | Cysteinylglycine | 2.39914 | -2.56133 | 0.00413 | down |
|  | D-Cysteine | 2.51111 | -1.58970 | 0.00474 | down |
|  | Indirubin | 2.45198 | -1.62383 | 0.00520 | down |
|  | Aflatoxin M1 | 2.73349 | -1.32577 | 0.00683 | down |
|  | 2-methyl-2,3,4,5-tetrahydro-1,5-benzoxazepin-4-one | 2.71076 | 1.41561 | 0.00827 | up |
|  | L-Glutathione oxidized | 1.40378 | 1.13065 | 0.00844 | up |
|  | Υ-Glutamylcysteine | 2.52883 | -1.57220 | 0.01181 | down |
|  | Biliverdin | 2.82158 | -1.32689 | 0.01243 | down |
|  | Bilirubin | 2.45463 | -1.31286 | 0.01831 | down |
|  | 5-Hydroxytryptophan | 3.35744 | 1.49181 | 0.02026 | up |
|  | ACar 19:2 | 1.94564 | 2.53912 | 0.02132 | up |
|  | 2-[3-methyl-2-(methylimino)-4-oxo-1,3-thiazolan-5-yl]acetic acid | 1.34311 | 1.13641 | 0.02233 | up |
|  | ACar 17:1 | 1.73579 | 1.70602 | 0.02379 | up |
|  | PC (14:1e/5:0) | 2.16919 | -1.35600 | 0.02590 | down |
|  | ACar 19:1 | 1.68300 | 2.21020 | 0.03144 | up |
|  | ACar 17:2 | 2.01003 | 1.33553 | 0.03453 | up |
|  | N-Formylkynurenine | 2.19769 | 1.64216 | 0.03479 | up |
|  | Epigallocatechin | 1.33519 | 1.02394 | 0.03556 | up |
|  | L-Ascorbate | 2.11519 | -1.52594 | 0.03970 | down |
|  | ACar 20:2 | 1.94398 | 1.31547 | 0.04389 | up |
|  | ACar 21:2 | 2.01509 | 3.70132 | 0.04951 | up |

*^a^*LSe: the selenium deficient group, SSe: the selenium supplement group, NEG: negative ion mode, POS: positive ion mode, VIP: variable importance in projection, Log_2_FC**:** Log_2_Fold change.
